# Global Expanded Nutrient Supply (GENuS) Model: A New Method for Estimating the Global Dietary Supply of Nutrients

**DOI:** 10.1371/journal.pone.0146976

**Published:** 2016-01-25

**Authors:** Matthew R. Smith, Renata Micha, Christopher D. Golden, Dariush Mozaffarian, Samuel S. Myers

**Affiliations:** 1 Department of Environmental Health, Harvard T H Chan School of Public Health, Boston, Massachusetts, United States of America; 2 Friedman School of Nutrition Science & Policy, Tufts University, Boston, Massachusetts, United States of America; 3 Department of Food Science and Human Nutrition, Agricultural University of Athens, Athens, Greece; 4 Center for Health and the Global Environment, Harvard T H Chan School of Public Health, Boston, Massachusetts, United States of America; 5 Harvard University Center for the Environment, Cambridge, Massachusetts, United States of America; Indiana University School of Medicine, UNITED STATES

## Abstract

Insufficient data exist for accurate estimation of global nutrient supplies. Commonly used global datasets contain key weaknesses: 1) data with global coverage, such as the FAO food balance sheets, lack specific information about many individual foods and no information on micronutrient supplies nor heterogeneity among subnational populations, while 2) household surveys provide a closer approximation of consumption, but are often not nationally representative, do not commonly capture many foods consumed outside of the home, and only provide adequate information for a few select populations. Here, we attempt to improve upon these datasets by constructing a new model—the Global Expanded Nutrient Supply (GENuS) model—to estimate nutrient availabilities for 23 individual nutrients across 225 food categories for thirty-four age-sex groups in nearly all countries. Furthermore, the model provides historical trends in dietary nutritional supplies at the national level using data from 1961–2011. We determine supplies of edible food by expanding the food balance sheet data using FAO production and trade data to increase food supply estimates from 98 to 221 food groups, and then estimate the proportion of major cereals being processed to flours to increase to 225. Next, we estimate intake among twenty-six demographic groups (ages 20+, both sexes) in each country by using data taken from the Global Dietary Database, which uses nationally representative surveys to relate national averages of food consumption to individual age and sex-groups; for children and adolescents where GDD data does not yet exist, average calorie-adjusted amounts are assumed. Finally, we match food supplies with nutrient densities from regional food composition tables to estimate nutrient supplies, running Monte Carlo simulations to find the range of potential nutrient supplies provided by the diet. To validate our new method, we compare the GENuS estimates of nutrient supplies against independent estimates by the USDA for historical US nutrition and find very good agreement for 21 of 23 nutrients, though sodium and dietary fiber will require further improvement.

## Introduction

Major trends in human nutrition are challenging to track at regional or global scales due to a lack of reliable and accessible data. Individual intake data, the optimal dataset for these types of studies, are generally lacking in the low-income developing countries for which they are most needed. Accurately measuring large-scale patterns such as the global nutrition transition to more processed and packaged foods or monitoring the decline in global nutritional deficiencies of vitamin A or folate requires the collection of hundreds of national surveys or clinical assessments to gain information on status for a given year. Lacking those resources, many studies on global nutrition rely on the FAO food balance sheets [1–5], which do not estimate consumption but crude national availability of many foods and selected nutrients including energy, fat, and protein. A major advantage of these FAO data is the provision of global coverage of food supplies at the country level since 1961. Yet, food balance sheets also have some important drawbacks, including a lack of specificity about many individual foods important to the diet, such as most fruits and vegetables, and no information about micronutrient contents. Furthermore, they use the so-called “subtraction” or “residual” method, where food supplies, rather than actual intake, are estimated by summing all domestic food supplies produced and traded by countries and removing those used for other applications other than human consumption. Finally, they only provide information at the level of total average supply per capita, which do not permit assessment of differences in intakes by age or sex.

Other studies have used household budget or consumption surveys to examine food or nutrient purchasing and estimate true intake [6–8]. However, only a few countries perform regular surveys, and most do not provide adequately comprehensive or accurate information to determine nutrient status [9]. To address these issues, additional studies have performed meta-analyses and compiled dietary surveys or biomarker analyses for specific nutrients and used Bayesian statistical models and FAO availability data to estimate intake values for countries or years with limited data [10–12]. These meta-studies provide the benefit of global coverage, as well as a closer approximation of true intake than FBS alone. Yet, they have only looked at coarse temporal trends of food group intake since 1990 and have not yet investigated most dietary micronutrient supplies.

We sought to overcome many of these limitations by constructing the GENuS model, which expanded on the broad and historical data of the food balance sheets to include additional food types, and matched them with nutritional information to build a global and historical nutrient supply database.

## GENuS Model Description

### Use and Expansion of Food Balance Sheets

**Fig 1:**
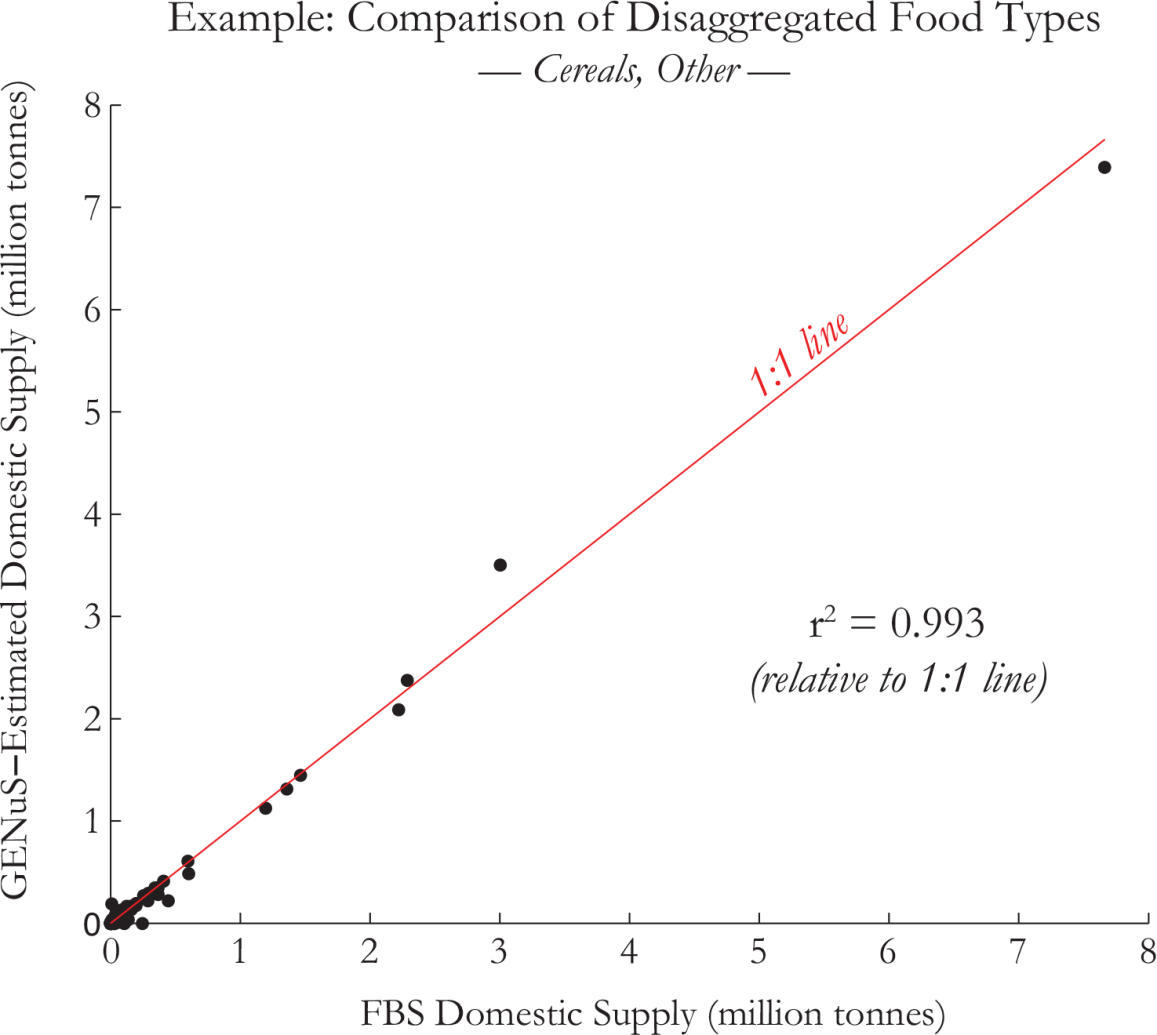
GENuS = FBS Disaggregation Comparison. Example of a comparison of FBS domestic supply values for all countries with summed GENuS model estimates. GENuS estimates are calculated by summing the supplies of the constituent foods (“Buckwheat”, “Fonio”, “Triticale”, “Mixed Grain”, “Popcorn”, “Quinoa”, “Canary Seed”, and “Cereals, nes (not elsewhere specified)”) and comparing them to the values supplied in the FAO food balance sheets listed as “Cereals, Other”.

FAO food balance sheets (FBS) report data on production, trade, and utilization of 98 food commodities from 1961 through 2011 for 175 current countries [13]. More importantly, they also provide estimates of daily food supplies per capita for many primary commodities that we used as direct inputs into our model. However, the FBS also include food categories that are broad (e.g. "Cereals, other", "Fruits, other") and comprise many sub-groups that are each important individual sources of nutrients to the diet, but the FBS do not include estimates of the supplies of these sub-groups individually. Therefore, we sought to disaggregate these groups down to the sub-groups by replicating the FBS methodology and using FAO production and trade data, listed elsewhere [14], for all constituent foods underneath each broad food group (e.g. "Apricots", "Plums", "Mangoes", etc. for "Fruits, other"). For each, we added the production amounts plus imports minus exports to calculate their domestic supply, and if necessary, converted the weights of any processed food back to its primary equivalent using conversion factors supplied by the FAO (e.g. "Dry Apricots" back to "Apricots") [15]. To verify the precision of this method, we summed the domestic supply values for each of these individual food types calculated separately by the model (e.g. "Apricots", "Plums", etc.) and compared them to the totals for the food category from the FBS ("Fruits, other"), and found that they generally correspond very well; the r^2^ values relative to a 1:1 line between the GENuS model estimates and FBS domestic supplies are shown in Table 1, with an example showing the correlation and r^2^ for “Cereals, other” in Fig 1. In the case of offals, the regressions using this method were especially poor, so we found a more accurate estimation by recalculating the offal weight using weight of meat. To do this, FAO factors were first used to convert the dressed carcass weight of meat back to live weight for each animal type, and additional conversion factors were then used to convert live weight to offal weight [15]. Other categories (e.g., “Oilcrops, other”, “Sweeteners, other”) also had particularly poor correlation after disaggregation, though these categories did not contain much nutritional variability within their groups, and we were able to leave them in their aggregated state.

**Table 1:** GENuS-FBS Data Correlation. r-squared values relative to a 1:1 line between the GENuS model estimates and FBS domestic supplies. An example of their formulation is shown in Fig 1.

Commodity category	r^2^	Commodity category, cont.	r^2^
Cereals, Other	0.993	Spices, Other	0.999
Roots, Other	0.999	Meat, Other	0.994
Pulses, Other	0.997	Offals, Edible	0.973
Treenuts	0.994	Butter, Ghee	0.998
Vegetables, Other	0.998	Eggs	1.000
Fruit, Other	0.999	Milk–Excluding Butter	0.984

We then converted between domestic supply and food supply, which removed the amount of each commodity that was allocated to non-food uses (e.g. "Feed", "Seed", "Processing", "Waste") or was held as an in-country stock. However, data on changes of stocks or non-food uses were not supplied down to the level of individual food type. Therefore, we used the ratio of domestic supply to food supply for the broader FBS category and applied it to each of the constituent foods. The resulting disaggregated dataset describes the food supplies per capita in 175 nations for 221 food commodities. A comparison of GENuS and FBS is shown in Table 2. These foods are meant to comprise the entire diet, thereby providing a comprehensive assessment of dietary and nutritional supplies. A full list of foods and their corresponding FBS categories are shown in the S1 Table.

**Table 2:** FAO Food Balance Sheet–GENuS Dataset Comparison.

	FAO Food Balance Sheets	GENuS Dataset
**Number of individual food commodities**	98	225
**Measured quantity**	Farm, carcass, or fresh catch weight	Edible weight
**Years covered**	1961–2011	1961–2011
**Countries covered**	175	152 (23 excluded due to poor data reporting)
**Global population covered**	100%	95.5%
**Nutrient supplies estimated**	Food energy, protein, fat	Food energy, protein, fat, carbohydrates, dietary fiber, calcium, vitamin C, vitamin A, folate, iron, zinc, copper, sodium, potassium, phosphorus, thiamine, riboflavin, niacin, vitamin B6, magnesium, saturated/monounsaturated/polyunsaturated fatty acids

### Fruit and Vegetable Food Supply Corrections

For most foods, the disaggregation above provided sufficiently detailed information to assess their nutritional value. However, we found that FAO data on fruit and vegetable supplies for many developing countries were notably sparse, with thirty-nine countries having greater than seventy five percent of their most recent fruit or vegetable domestic supply listed as “Fruit, fresh nes (not elsewhere specified)”, “Fruit, tropical fresh nes”, or “Vegetables, fresh nes”. Because the nutritional density of fruits and vegetables can vary greatly across type, this posed a problem for calculating precise nutrient supplies.

The FAO classifies data as “nes” for two reasons: 1) countries are consuming and reporting foods that are not recorded explicitly by the FAO, or 2) the FAO receives discordant data from multiple sources (i.e., production data on individual fruits that do not match data from a separate source that reports production of total fruit) and the difference between the two is listed as “nes”. Fortunately, we can distinguish between these cases by analyzing FAO data tags. The FAO accompanies each piece of released data with a tag that denotes whether the data point is official, unofficial, estimated or imputed. If a country were simply eating and reporting a considerable amount of a fruit or vegetable that does not fit into another FAO category, it would be listed as “Official Data” by the FAO. If a country were using the “nes” category to report the balance of mismatched data from multiple sources, the data would be tagged as an “FAO estimate” or would be imputed from a previous year’s estimates. We find that only two of those thirty-nine countries report any of their “nes” fruit or vegetable supplies in 2011 as official data, while thirty-six of the countries report the data as estimated or imputed from previous estimates, and only one reports the data as unofficial.

For those countries with high proportions of estimated or imputed “nes” fruits and vegetables, we attempted to solve this ambiguity in two ways. First, and most directly, we identified six countries among this group that have recent and publicly available agricultural censuses—Angola (2012) [16], Guinea-Bissau (2009) [17], Laos (1998–9) [18], Mozambique (2011) [19], Nepal (2010) [20], and Tanzania (2007–8) [21]. To include these countries in our assessment of current nutritional supplies, we took their total FAO-derived fruit and vegetable production for the most recent year and redistributed them based on the percentages of individual fruit and vegetable production calculated from the censuses; previous years’ data were omitted for these countries only. For the rest of the countries that had estimated or imputed data, we distributed the “nes” amounts between the individual fruit and vegetable groups proportionately based on their supply values that we had estimated using the FAO production and trade statistics. When a country reported fruit or vegetable supplies in few or no other categories, which we arbitrarily set at five or fewer per food group, we omitted them from our database due to concerns about the precision of their reporting, removing twenty-three countries, and leaving 152 countries in total that represent 95.5% of the global population.

### Calculating Edible Portions

After estimating the total food supply by type, we then converted total commodity weight to edible weight. This correction mainly applied to fruits, vegetables, nuts, seeds, and animal products for which the FAO values include the weight of non-edible portions such as bones, shells, and peels. FAO agricultural commodity data are reported in terms of primary equivalents, which represent the sum of the weight of the primary commodity plus any processed versions of that product converted back to the weight of its original commodity. Data on livestock primary products are given in dressed carcass weight, which reflects the bone-in weight after slaughter and initial butchering, while seafood data typically are given as the weight of fresh or landed catches. However, in order to calculate the nutrients supplied by foods, which are often listed as nutrient density per hundred grams of edible portion, we needed to convert the FAO primary weights to edible weights.

The first step for meat and seafood was to convert the slaughterhouse or fresh weight to retail weight. For livestock, this reflects the removal of some bones and fat for sale or consumption; for seafood, this represents gutting, filleting or shelling the fresh catch. We used USDA conversion factors for individual livestock types (bovine, pig, poultry, and pig/goat) [22], and FAO factors for individual seafood types [23], of which we used the average value to match with the FAO fish and shellfish categories that we used (freshwater, demersal, pelagic and other fishes; crustaceans, cephalopods, molluscs). We did not account for similar losses between farm and retail for agricultural commodities because FAO already calculates them using estimates made by local trade groups and experts.

Finally, we converted retail weights to edible weights by estimating and subtracting the discarded percentage (by weight) for each food, such as fruit peels and nut shells, using the average discarded values listed for that category as given by the USDA Nutrient Database [24]. The discarded percentages by weight that we used are listed in S1 Table.

### Processed Foods

Refined grains and cheese are widely consumed processed foods that have a distinct nutritional profile from their original commodity. We addressed these specially to account for their potential influence on nutrient supplies.

For most major cereals, milling and refining grains has a major influence on their final nutrient profiles, often removing micronutrients in the process. Therefore, we estimated the amount of processed and refined grain for many cereals—wheat, maize, millet and sorghum—using the regional processing estimates from Wessells et al. (2012) [25]. Rice was excluded from this treatment because it was reported by the FAO in its milled weight, and most rice consumed globally is milled [26,27]. For wheat, maize, millet and sorghum, we converted their weight to flour by multiplying the unprocessed grain weight by the global average grain-to-flour weight difference given by the FAO [15]. The remainder of unprocessed grain was assumed to have the nutrient density of the whole grain or whole-grain flour. The addition of refined cereal grain flours to the model increased the number of estimated commodities to 225.

For milk, the FAO only reports on milk supplies, which are meant to include all dairy products that are produced from milk as well. However, because cheese is an important food staple globally and the process of making cheese greatly changes its nutrient profile relative to raw milk, we included the nutrient densities for cheeses along with whole milk within our “Milk” category. We did so by converting cheeses back to their whole milk equivalent by dividing their nutrient densities by the appropriate USDA milkfat conversion factor [22]. For especially soft Indian cheeses (i.e., channa and khoa), we normalized their nutrient densities to obtain the milkfat content of Indian whole milk.

### Estimating Nutrient Supplies

After estimating the edible food supplies in each country, we then matched foods with nutritional densities from food composition tables to determine the nutrient supply per capita. Six national and regional nutrient composition tables were used: West Africa [28], Latin America [29], Southeast Asia [30], Northeast Asia [31], the United States [24], and India [32]. We applied the West African table to all sub-Saharan African countries, and for those foods without values we complemented the dataset with an older FAO African table [33]; if neither had values for a given food, the USDA values were used. For each table, we individually matched each of our food categories with their most comparable foods. Countries were assigned a food composition table that was regionally appropriate or, failing that, were assigned the USDA nutrition table. Major areas that were not covered by the regional databases were the Middle East, North Africa and Europe.

We restricted the number of nutrients analyzed to 23: calories, fat, protein, carbohydrates, dietary fiber, vitamin C, vitamin A, folate, thiamin, riboflavin, niacin, total B6, calcium, iron, zinc, potassium, copper, magnesium, selenium, phosphorus, saturated fatty acids, monounsaturated fatty acids, and polyunsaturated fatty acids. Nutrients that were found in two or more tables were included, though only six (folate, total B6, magnesium, saturated FA, monounsaturated FA, polyunsaturated FA) were found in three or fewer. Most nutrients were found in all six tables.

If multiple foods described a specific category equally well (e.g., both “Russet potato” and “Red potato” for FBS’s “Potatoes”), all foods were included for later steps. The foods chosen to represent each category were restricted to the least processed and uncooked versions to preclude addition of other ingredients. At this stage, fortified versions of foods were also omitted to help compare across countries with different fortification policies, though they are addressed in later steps. If a regional table did not include information about a certain food, the USDA value is included instead if it exists. In the absence of a USDA value, a global average of all tables was used.

To calculate the range of possible nutrient supplies, we used Monte Carlo simulations to estimate the range of nutrients associated with edible food supplies. For each of a thousand iterations, we randomly chose one from among the foods under each food category to be representative of its nutritional density. For example, when estimating the food energy contribution from potatoes, for which there are three USDA entries (“Russet”, “Red” and “White” potatoes) each with a corresponding food energy value (79, 69 and 70 kcal per 100 grams), we randomly chose from among the three to describe the energy density of potatoes on each iteration. Each representative food’s nutrient information was then multiplied by the food supply amounts and summed to calculate nutrient availabilities per capita on each run. For potatoes, we multiplied its randomly chosen energy density, 70 kcal/100 g for example, by its consumption for that country and year (114 g/day in the United States in 2011), to arrive at a subtotal of 80 kcal per day supplied by potatoes on that iteration. This was repeated for all food groups and summed to calculate total nutrient supplies in each country and year. The results for all 1,000 iterations were tabulated to determine medians and 95% uncertainty interval values.

### Fortification

Vitamin and mineral fortification are major sources of some nutrients in many countries with mandatory or voluntary governmental or industrial programs. To estimate their contribution to nutrient supplies for the most recent year only, we compiled a database of current regulatory and voluntary fortification guidelines using several data sources that describe common levels of nutrients added per kilogram of product (S1 File). Fortified nutrients studied and their food vehicles are shown in S2 Table.

When a range of possible fortification amounts were given, we chose the lower bound, assuming that a percentage of the added nutrient will continue to be lost through processing, storage and transport [34], and that the lower value is a better estimate of the fortified nutrient in the final consumed product.

For cereal flours and meals in particular, often they are produced at home or in local mills, where fortificants are rarely added due to lack of regulation enforcement or insufficient supply to rural areas, especially in developing countries. Therefore, we used estimates of the amount of cereal flours produced in industrial mills as estimated by the Food Fortification Initiative [35], and only modeled the fortification of this proportion.

### Supplies by Age and Sex

Expanding on nationally averaged per capita nutrient supplies, we also calculated age and sex-specific supplies of food and nutrients for each country for the current year only across 26 age-sex groups (ages 20+ in five-year increments for both sexes) using dietary data taken from the Global Dietary Database (GDD) [36] on intakes across eleven broad food groups and nine nutrients for 187 countries, as well as averages for 21 regions and worldwide. A more detailed description of the GDD methodology may be found in previous studies [10, 37–39]. GDD data were built by first aggregating and reanalyzing individual-level intake data of major foods and nutrients that are related to non-communicable diseases in specific age/sex groups worldwide, rather than nutrient supplies, using nationally representative data where available. Data from primary sources were then combined with food balance sheets available in all nations and years. A Bayesian hierarchical model was used to estimate mean food and nutrient intake and associated uncertainty for each age-sex-country-year stratum accounting for differences in intakes versus availability, survey methods and representativeness, and sampling and modeling uncertainty.

We used food and calorie supply estimates from GENuS to normalize each country's average per-capita supplies for each of the 225 foods to a 2,000 calorie diet to further match the GDD estimates. For example, if Country X has a per capita supply of apples of 30 g/day and total food energy supply of 3,000 kcal/day, we calculate a 2,000-calorie-adjusted apple supply of 20 g/day (30 g/day * 2,000/3,000). Next, we matched each GENuS food to a corresponding GDD food category (listed in S1 Table). In our example, apples would be paired with GDD’s “Fruit” category. For oils and oilseeds, there were no direct GDD intake estimates, so we instead paired them with the specific fatty acid that we assumed to best represent a similar pattern of intake: plant omega-3 (rape/mustard seed), polyunsaturated fatty acids (cottonseed, groundnut, soybean, sunflower, olive, rice bran, maize germ, sesame seed, and other), saturated fatty acids (palm kernel, palm, coconut, animal fats), and seafood omega-3 (fish oils). For some of the remaining foods without a matching food or nutrient (representing 14% of global calories)—starchy vegetables, sweeteners, eggs—we assumed that all age-sex groups had the same energy-adjusted intake. For other foods that are mainly consumed by adults—alcohol, stimulants and spices—we assumed zero consumption for children and adolescents.

Next, for GENuS foods that had a corresponding GDD food or nutrient category, we used the ratio between the food intake for each GDD age-sex group and the population-wide average value for each country, and multiplied that ratio by the GENuS energy-adjusted food supplies to construct supply estimates by age and sex. For apples in Country X, we first calculate how much more or less each age-sex group consumes relative to the population-weighted national average; if women aged 50–54 consume 10% more fruit than average, we estimate that the supply of apples is 10% greater than the calorie-adjusted average, as calculated above (20 g/day): 22 g/day. To estimate energy-adjusted food supplies for children and adolescents (ages <20) for which there are currently no GDD estimates, we simply assumed that they consumed the average value (children/adolescents in Country X have an average calorie-adjusted supply of apples: 20 g/day). Age-sex-specific values are calculated so that, when weighted by population size of each age-sex group in each country, they will total the national per capita nutrient supply. The next iteration of GDD will have estimates for children and adolescents as well, which will be incorporated when available.

We then converted energy-adjusted food intakes back to absolute intakes. Here, we assumed that each age-sex group received calories in proportion to their needs, and we used the country's total per-capita calorie supply, coupled with estimates of energy requirements by age and sex [40] and each country's population demographics from the 2012 UN World Population Prospects [41], to estimate the caloric supply for each age- and sex-group. Then, we used this age-sex-specific calorie supply to re-correct the energy-adjusted food estimates back to absolute supplies for all foods and countries. If we return to the example of Country X, we would use the country’s demographic mix to estimate that the country’s average per capita dietary energy needs are 2,500 kcal/person/day. Because we’ve previously calculated that the average energy supply is 3,000 kcal/person/day, we find that the energy supply exceeds needs by 20%. If we assume that this percentage applies equally to all age-sex groups and the dietary needs for women aged 50–54 is 2,200 kcal/day, then we estimate their supply is 20% higher: 2,640 kcal/day. Therefore, the unadjusted supply of apples would be 22 g/day * (2,640/2,000): 29 g/day.

Then, as we did for national average supplies, we matched each age- and sex-specific food supply to its corresponding nutrient density, and multiplied and summed them across all food types to calculate age- and sex-specific nutrient supplies.

### Accessible Data

GENuS datasets, both nationally averaged and for specific age-sex groups are free and publicly available at http://projects.iq.harvard.edu/pha/genus. Available datasets are: nationally averaged individual food [42] and nutrient supplies [43] by country and year, total nutrient supplies in 2011 including fortification [44], nutrient supplies from each food by country in 2011 [45], individual food supplies by country and age-sex group for 2011 [46], nutrient supplies (summed across all food types) by country and age-sex group for 2011 both with and without the contribution of fortification [47,48]. Where applicable, all datasets include median and 95% uncertainty intervals from the Monte Carlo estimation algorithm.

## Validation

To validate our model, we compared our GENuS model predictions against an established and long-running historical dataset on food and nutrient supplies in the US, from the USDA’s Center for Nutrition Policy and Promotion [49]. The USDA calculates food supplies using a similar methodology as the FAO, assuming that food commodities that have not been allocated to other uses are available as food. However, unlike the FAO and GENuS, the USDA has data on food supplies for over 400 individual commodities that are specifically chosen to record the breadth of the US diet; of the 225 foods included in GENuS model, Americans only eat more than a gram per year of ~140 of them, and they include broad categories (e.g., “Milk” or “Tomatoes”) which include many processed forms. By seeing whether there is general agreement between the GENuS output and the more detailed USDA dataset, we can gain confidence that our model may be applied to countries without corroborating data.

**Fig 2:**
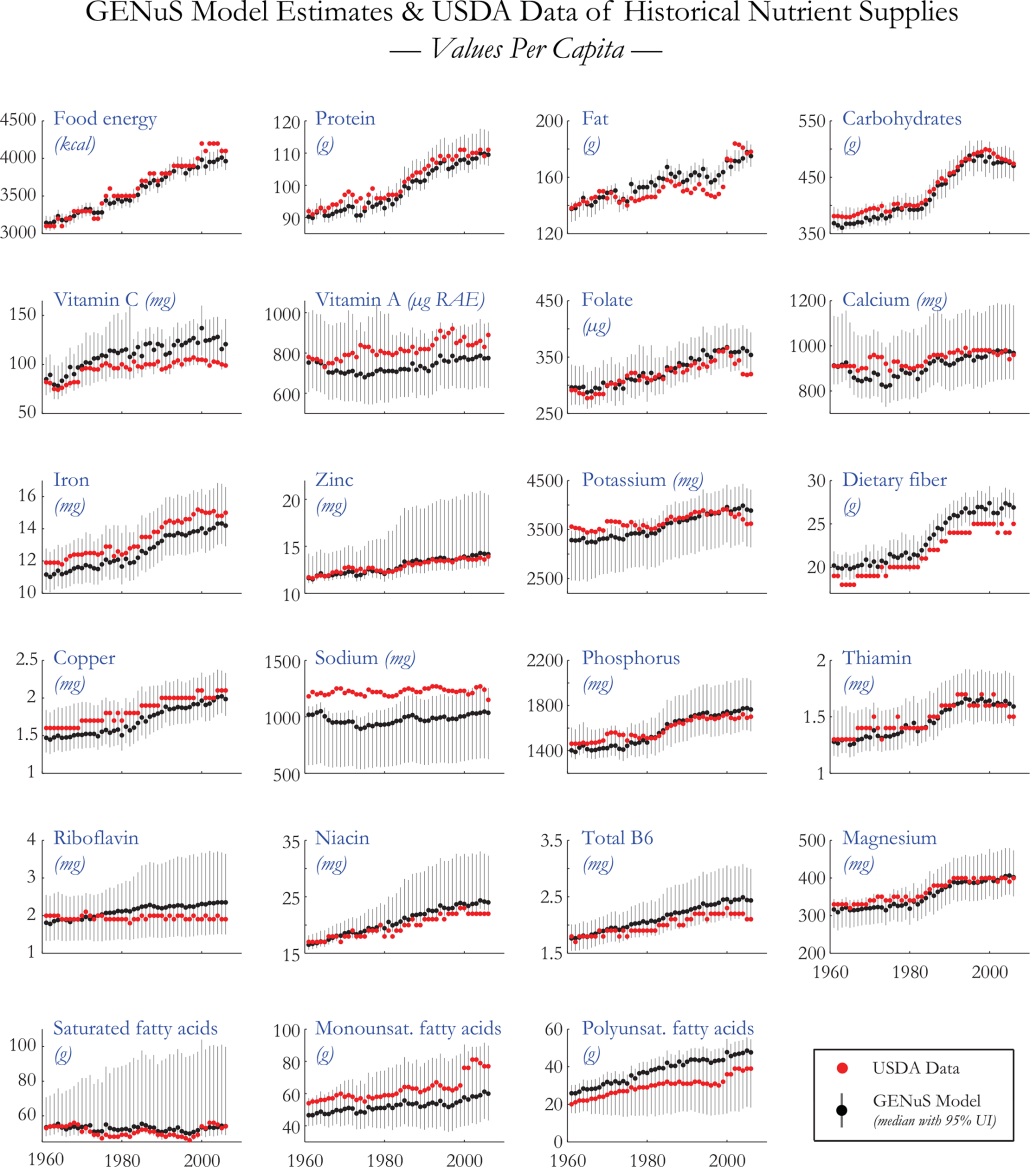
GENuS-USDA Nutrient Supply Comparison. Comparisons of GENuS model estimates and USDA nutrient supplies (data available in S1 Data). To compare GENuS estimates with historical data (where fortification levels are not estimated), the USDA supplies shown exclude nutrients added through fortification. A comparison of the USDA values and the GENuS model including fortification is found in Table 3.

The results of the comparison are shown in Fig 2. We found reasonable agreement between GENuS estimates and USDA data for many nutrients. For some, such as protein and carbohydrates, the range of possible GENuS outputs is very narrow and tracks the USDA data very well. Though, for many micronutrients there was a broader range of possible GENuS outputs due to the heterogeneity of nutrient densities of foods within GENuS categories. However, even those nutrients with the broadest range in the US, such as vitamin A (95% UI: 624–908 μg RAE), are capable of being distinguished from countries with a greater risk for deficiency from dietary sources, such as Bangladesh (50–162 μg RAE) or India (57–500 μg RAE).

We also tested the precision of our fortification estimates by applying the full GENuS model including fortification to the most recent year for which the USDA has estimated total nutrient supplies, 2010. The comparison between these two is shown in Table 3. We found that the agreement between the GENuS and USDA values is also consistently good, with all USDA values falling within the 95% uncertainty interval of the model.

**Table 3:** GENuS-USDA Data Comparison for Fortified Nutrients. USDA [50] vs. full GENuS estimates of 2010 supplies of nutrients that are commonly fortified.

	USDA	GENuS (incl. fortification)
		*Median (95% UI)*
Iron (mg)	24.6	22.5 (21.3–24.6)
Folate (μg)	889	874.3 (844.8–911.8)
Vitamin A (μg RAE)	920	934.1 (796.1–1078.4)
Thiamin (mg)	2.9	2.9 (2.7–3.1)
Riboflavin (mg)	3	3.1 (2.3–4.2)
Niacin (mg)	34	33.4 (32.4–40.1)

The nutrients for which there is more limited agreement in our model are sodium and dietary fiber. For sodium, we underestimate the level of sodium supply by up to 20% compared with the USDA, which we attribute to our exclusion of added salt in foods in GENuS which are included in many processed foods included in the USDA estimates. Because the FBS provide no information on added salt in the food supply, and because added salt contributes so heavily to the global sodium intake [11], GENuS is only suitable for estimating sodium supplied directly from foods and not total dietary sodium intake. For dietary fiber, USDA estimates are consistently slightly below our model predictions, presumably because we are not capturing some processed foods, such as fruit juices, that are lower in dietary fiber than their whole food equivalent. Therefore, GENuS predictions provide an upper bound on dietary fiber supplies, and should continue to be refined.

Because we only have available comparable data on nutrient supplies from one country with high-quality statistical accounting—the United States—this exercise only demonstrates the validity of our underlying methodology. However, a major limitation of the validation and GENuS in general is the quality of the agricultural statistics supplied to the FAO upon which GENuS is based. Therefore, our estimates of food and nutrient supplies can only be as accurate as the available data, which may be compromised in developing countries with poorer statistical accounting.

## Limitations: Supply vs. Consumption

A potential drawback of our nutrient estimates is that they provide information only on supplies, not true consumption of each food at the individual level. It has been found in previous studies that the FBS do not accurately predict, and often overestimate, true consumption, and the gap between the two can vary substantially by food group, world region, and other characteristics [51]. This problem is further exacerbated by a lack of consistency as to whether wild or locally grown foods are included in consumption measures.

Similarly, food balance sheets provide information in units of primary equivalents, and return all processed foods back to the weight of its original commodity. This too likely causes an overestimation of true nutrient availabilities due to nutrient losses through food processing, food waste in the home, and cooking and preparation. Previous studies have shown that these losses can result in overestimates of 20–180% between FBS and consumption rates [52].

These two factors taken together allow us to use this method only as an assessment of the food and dietary nutrient supplies of each country, not consumption itself. Because we are using supplies instead of consumption and assuming higher-than-true intakes, we are likely providing a lower bound on the sizes of populations that are at risk for deficiency. However, food supplies provide a theoretical upper limit for potential intake for a country if spoilage and food waste were minimized. Until other direct methods of accounting of consumption, such as household surveys, are capable of providing the breadth of coverage of the FBS, the balance sheets will remain the best available tool for global coverage, though caution must be applied to their interpretation.

## Using the GENuS Model

The GENuS model provides a novel method for estimating nutrient supplies, extrapolating the only current global long-term dataset—food balance sheets—paired with regional nutrient density tables to produce a powerful tool for nutritional studies. It has three main strengths. First, it provides a long-term dataset of national nutrient availabilities so that multi-year or decadal trends and progress can be measured. Second, it takes a much deeper look at the current nutritional supplies, breaking apart national averages to reveal patterns of nutritional availability by 34 age and sex groups, as well as the impact of fortification in meeting nutritional adequacy. Finally, because GENuS describes the entire diet as comprised by individual foods, it allows us to examine how changing access to any one of these foods can affect nutritional adequacy by country. These combined novel features unlock many previously unavailable analyses.

Global environmental change is extensive and accelerating, and its broad reach has the potential to affect the yield and nutritional quality of many foods important in the diet. Many examples of potential effects on diet and nutrition have been recently explored. Anthropogenic increases in atmospheric CO_2_ levels have been shown to reduce the nutritional content of major grain and legume crops [53], while increases in CO_2_ levels and global temperatures appear to have opposing impacts on crop yields [54]. Sustained losses of animal pollinators due to environmental pressures could reduce production of pollinator-reliant crops, such as fruits and vegetables, and drive significant increases in chronic disease and malnutrition [55]. Also, degradation of marine fisheries through overfishing and climate change is currently affecting the delivery of protein and micronutrients to vulnerable populations in many developing countries [56].

In order to more fully assess the impact of these and other environmental drivers on global diets, we first need to know populations’ reliance on affected foods, which has been a missing link in global analyses until now. The GENuS dataset fills this gap by providing a model of global diets and the importance of individual foods in achieving adequate nutrition. By providing quantified estimates of the roles of individual foods in the nutritional status of different populations, GENuS is a potentially powerful tool to understand and tackle major issues in global nutrition and food security.

## Conclusions

The GENuS model provides a new and detailed method to estimate supplies of key micro- and macronutrients globally, based on 225 food commodities within 152 countries over the past 50 years. This model also allows better estimation of heterogeneity in global nutrition by analyzing nutrient supplies across 34 age and sex groups. Because this method calculates nutrient supplies down to the individual food group, it is a more powerful tool for calculating how changes in access to particular foods or changes of nutrient composition of those foods would potentially impact intake of specific nutrients for target populations and the overall adequacy of their nutrition. This not only allows researchers or policymakers to find those most vulnerable for nutrient deficiency or malnutrition, but also to know which foods supply those nutrients, and which countries would be vulnerable to changes in production or access to these foods. For example, GENuS can allow, for the first time, robust estimation of the nutrient supply consequences of varying threats to different foods due to climate change, soil degradation, drought, loss of pollinators, and other modern challenges. By using food supply data over the past half-century, we have the unique ability to attain nearly global coverage to measure worldwide trends. Because of its scope and detail, the GENuS model can provide a useful and versatile method for monitoring global nutrition and health in a rapidly changing world.

## Supporting Information

S1 Data GENuS-USDA Nutrient Supply Comparison Data. Nutrient supply estimates from GENuS compared with corresponding USDA nutrient supply estimates since 1961 (excluding fortification).
(XLS)

S1 File Fortified foods and country-specific guidelines. (DOCX)

S1 Table GENuS Foods and Associated Categories. Associated GDD food groups and non-edible (discarded) percentage for each food studied
(DOCX)

S2 Table Fortified nutrients studied and their associated food vehicles. (DOCX)
