# Supplementary material for: Global Expanded Nutrient Supply (GENuS) Model: A New Method for Estimating the Global Dietary Supply of Nutrients
Source: PLoS One. 2016 Jan 25;11(1):e0146976. doi: 10.1371/journal.pone.0146976 (PMC4726504; doi:10.1371/journal.pone.0146976)
Supplement: S1 File — (DOCX) [file pone.0146976.s002.docx]

**FORTIFICATION FOR MOST COUNTRIES**

| *Country/region:* | Global |
| --- | --- |
| *Nutrients:* | Iron, folic acid, zinc, vitamin A |
| *Foods:* | Wheat flour, corn meal/flour, rice |
| *Source:* | Food Fortification Initiative, Country fortification profiles. 2015; Available: http://www.ffinetwork.org/country_profiles/ |

| *Country/region:* | Global |
| --- | --- |
| *Nutrients:* | Iron, folic acid, thiamin, riboflavin, niacin, vitamin A |
| *Foods:* | Wheat flour, corn meal/flour, rice, milk, margarine, cooking oil, sugar |
| *Source:* | Mandatory food enrichment. NutriView special issue. Basel, Switzerland: Roche Vitamins Europe; 2003. |

| *Country/region:* | Africa |
| --- | --- |
| *Nutrients:* | Thiamin, riboflavin, niacin, vitamin A |
| *Foods:* | Wheat flour, corn meal/flour, rice, milk, margarine, cooking oil, sugar |
| *Source:* | Strategic Alliance for the Fortification of Oil, Food Fortification Legislation: Africa. 2007. Available: http://www.food-fortification.com/Files/Download/Resources/FF%20Laws%20Africa.pdf |

| *Country/region:* | Asia |
| --- | --- |
| *Nutrients:* | Thiamin, riboflavin, niacin, vitamin A |
| *Foods:* | Wheat flour, corn meal/flour, rice, milk, margarine, cooking oil, sugar |
| *Source:* | Strategic Alliance for the Fortification of Oil, Food Fortification Legislation: Africa. 2007. Available: http://www.food-fortification.com/Files/Download/Resources/FF%20Laws%20Asia.pdf |

| *Country/region:* | Latin America, North America, Caribbean |
| --- | --- |
| *Nutrients:* | Thiamin, riboflavin, niacin, vitamin A |
| *Foods:* | Wheat flour, corn meal/flour, rice, milk, margarine, cooking oil, sugar |
| *Source:* | Strategic Alliance for the Fortification of Oil, Food Fortification Legislation: Africa. 2007. Available: http://www.food-fortification.com/Files/Download/Resources/FF%20Laws%20LatinCarib.pdf |

**EXCEPTIONS FOR INDIVIDUAL COUNTRIES**

*Africa*

| *Country/region:* | Ghana |
| --- | --- |
| *Nutrients:* | Vitamin A |
| *Foods:* | Wheat flour, cooking oil |
| *Source:* | Nyumuah RO, Hoang TC, Amoaful EF, Agble R, Meyer M, Wirth JP, et al. Implementing large-scale food fortification in Ghana: Lessons learned. Food Nutr. Bull. 2012; 33(4) supplement: 5293–5300. |

| *Country/region:* | Uganda |
| --- | --- |
| *Nutrients:* | Folic acid, thiamin, riboflavin, niacin |
| *Foods:* | Wheat flour |
| *Source:* | Fortified wheat flour – specification. Uganda National Bureau of Standards. 2012. |

| *Country/region:* | Uganda |
| --- | --- |
| *Nutrients:* | Folic acid, thiamin, riboflavin, niacin |
| *Foods:* | Corn meal/flour |
| *Source:* | Fortified milled maize (corn) products – specification. Uganda National Bureau of Standards. 2012. |

| *Country/region:* | Uganda |
| --- | --- |
| *Nutrients:* | Vitamin A |
| *Foods:* | Sugar |
| *Source:* | Fortified sugar – specification. Uganda National Bureau of Standards. 2012. |

| *Country/region:* | Uganda |
| --- | --- |
| *Nutrients:* | Vitamin A |
| *Foods:* | Cooking oil, margarine |
| *Source:* | Fortified edible oils and fats – specification. Uganda National Bureau of Standards. 2012. |

| *Country/region:* | West African Economic and Monetary Union (UEMOA): Benin, Burkina Faso, Cote d’Ivoire, Guinea-Bissau, Mali, Niger, Senegal, Togo* |
| --- | --- |
| *Nutrients:* | Vitamin A |
| *Foods:* | Cooking oil |
| *Source:* | Sablah M, Klopp J, Steinberg D, Touaoro Z, Laillou A, Baker S. Thriving public-private partnership to fortify cooking oil in the West African Economic and Monetary Union (UEMOA) to control vitamin A deficiency: Faire Tache d’Huile en Afrique de l’Ouest. Food Nutr. Bull. 33(4) supplement: 5310–5320. |
| *** | *There is no standard fortification level for this initiative. We used the common value of 25,000 IU (7,500 μg RAE) used in Mali in all UEMOA countries.* |

| *Country/region:* | Zambia |
| --- | --- |
| *Nutrients:* | Iron, folic acid, zinc, thiamin, riboflavin, niacin |
| *Foods:* | Wheat flour, corn meal/flour |
| *Source:* | Fiedler JL, Levidini K, Zulu R, Kabaghe G, Tehinse J, Bermudez OI. Identifying Zambia’s industrial fortification options: Toward overcoming the food and nutrition gap-induced impasse. Food Nutr Bull 2013; 34(4): 480–500. |

*Latin America, North America, Caribbean, Europe*

| *Country/region:* | Belize |
| --- | --- |
| *Nutrients:* | Thiamin, riboflavin, niacin |
| *Foods:* | Wheat flour |
| *Source:* | Belize National Standard Specification for Wheat Flour (First Revision), BZS 2. 2007. |

| *Country/region:* | Brazil, Denmark |
| --- | --- |
| *Nutrients:* | Vitamin A |
| *Foods:* | Margarine, cooking oil |
| *Source:* | Lotfi M, Mannar MGV, Merx RJHM, van den Heuvel PN. Micronutrient Fortification of Foods: Current practices, research and opportunities. The Micronutrient Initiative/International Agricultural Centre. Ottowa. 1996. |

| *Country/region:* | CARICOM (Antigua & Barbuda, Bahamas, Barbardos, Dominica, Grenada, Guyana, Haiti, Jamaica, St. Kitts & Nevis, St. Lucia, St. Vincent & the Grenadines, Suriname, Trinidad & Tobago) |
| --- | --- |
| *Nutrients:* | Thiamin, riboflavin, niacin |
| *Foods:* | Wheat flour |
| *Source:* | Specification for wheat flour. Saint Lucia Bureau of Standards. 2014. |

| *Country/region:* | Chile |
| --- | --- |
| *Nutrients:* | Folic acid |
| *Foods:* | Wheat flour |
| *Source:* | Lancelloti CC. Analisis de los beneficios y riesgos de la fortificacion de harina de trigo con acido folico en Chile. Ph.D. Dissertation, Universitat de Les Illes Balears. 2014. Available: http://www.tdx.cat/bitstream/handle/10803/145979/tcaca1de1.pdf? sequence=1 |

| *Country/region:* | Dominican Republic |
| --- | --- |
| *Nutrients:* | Thiamin, riboflavin, niacin |
| *Foods:* | Wheat flour |
| *Source:* | Rao, M. Informe del Estudio Sobre el Programa de Fortificacion de Harina de Trigo en la Republica Dominicana. Presentation. Emory University. Jul. 2013. |

| *Country/region:* | Ecuador |
| --- | --- |
| *Nutrients:* | Thiamin, riboflavin, niacin |
| *Foods:* | Wheat flour |
| *Source:* | Lineamientos para la Implementacion del Sistema de Monitoreo Interno del Programa de Fortificacion de Harina de Trigo. Ministereo de Salud Publica del Ecuador. Coordinacion Nacional de Nutricion. 2011. |

| *Country/region:* | El Salvador |
| --- | --- |
| *Nutrients:* | Vitamin A, thiamin, riboflavin, niacin |
| *Foods:* | Corn meal/flour, sugar |
| *Source:* | Fortificacion de Alimentos. Especificaciones. Azucar, sal, harina de maiz nixtamalizado y pastas alimenticias. Reglamento Tecnico Salvadoreno. RTS 67.06.01:13. 2011. |

| *Country/region:* | Guatemala |
| --- | --- |
| *Nutrients:* | Thiamin, riboflavin, niacin |
| *Foods:* | Wheat flour |
| *Source:* | Consolidado de legislacion para fortificacion de alimentos. Comision Nacional para la Fortificacion Enriquecimiento y/o Equiparacion de Alimentos (CONAFOR), Republica de Guatemala. 2010. |

| *Country/region:* | Honduras |
| --- | --- |
| *Nutrients:* | Thiamin, riboflavin, niacin |
| *Foods:* | Wheat flour |
| *Source:* | Harinas. Harina de Trigo Fortificada. Especificaciones. Reglamento Tecnico Centroamericano. NSO RTCA 67.01.15:06. |

| *Country/region:* | Mexico |
| --- | --- |
| *Nutrients:* | Thiamin, riboflavin, niacin |
| *Foods:* | Corn meal/flour |
| *Source:* | Burton KE, Steele FM, Jefferies L, Pike OA, Dunn ML. Effect of Micronutrient Fortification on Nutritional and other Properties of Nixtamal Tortillas. Cereal Chem 2008; 85(1): 70–75. |

| *Country/region:* | United States |
| --- | --- |
| *Nutrients:* | Folic acid |
| *Foods:* | Wheat flour |
| *Source:* | Crider KS, Bailey LB, Berry RJ. Folic Acid Food Fortification – Its History, Effect, Concerns, and Future Directions. Nutrients; 3: 370–384. doi: 10.3390/nu3030370. |
