# Supplementary material for: Global Expanded Nutrient Supply (GENuS) Model: A New Method for Estimating the Global Dietary Supply of Nutrients
Source: PLoS One. 2016 Jan 25;11(1):e0146976. doi: 10.1371/journal.pone.0146976 (PMC4726504; doi:10.1371/journal.pone.0146976)
Supplement: S1 Table — Associated GDD food groups and non-edible (discarded) percentage for each food studied (DOCX) [file pone.0146976.s003.docx]

| Food | FBS Commodity Group | GDD Food Group | Discarded % by weight (USDA) |
| --- | --- | --- | --- |
| Wheat | Wheat | Whole grains | 0.0 |
| Rice (Milled Equivalent) | Rice (Milled Equivalent) | Whole grains | 0.0 |
| Barley | Barley | Whole grains | 0.0 |
| Maize | Maize | Whole grains | 0.0 |
| Rye | Rye | Whole grains | 0.0 |
| Oats | Oats | Whole grains | 0.0 |
| Millet | Millet | Whole grains | 0.0 |
| Sorghum | Sorghum | Whole grains | 0.0 |
| Buckwheat | Cereals, Other | Whole grains | 0.0 |
| Fonio | Cereals, Other | Whole grains | 0.0 |
| Triticale | Cereals, Other | Whole grains | 0.0 |
| Mixed grain | Cereals, Other | Whole grains | 0.0 |
| Cereals; nes | Cereals, Other | Whole grains | 0.0 |
| Popcorn | Cereals, Other | Whole grains | 0.0 |
| Quinoa | Cereals, Other | Whole grains | 0.0 |
| Canary seed | Cereals, Other | Whole grains | 0.0 |
| Cassava | Cassava | *none* | 16.0 |
| Potatoes | Potatoes | *none* | 25.0 |
| Sweet Potatoes | Sweet Potatoes | *none* | 28.0 |
| Yams | Roots, Other | *none* | 14.0 |
| Yautia (cocoyam) | Roots, Other | *none* | 14.0 |
| Taro (cocoyam) | Roots, Other | *none* | 14.0 |
| Roots and tubers; nes | Roots, Other | *none* | 19.2 |
| Flour of roots and tubers | Roots, Other | *none* | 0.0 |
| Sugar Cane | Sugar Cane | *none* | 0.0 |
| Sugar; Non-Centrifugal | Sugar; Non-Centrifugal | *none* | 0.0 |
| Sugar (Raw Equivalent) | Sugar (Raw Equivalent) | *none* | 0.0 |
| Sweeteners; Other | Sweeteners; Other | *none* | 0.0 |
| Honey | Honey | *none* | 0.0 |
| Beans | Beans | Legumes | 0.0 |
| Peas | Peas | Legumes | 0.0 |
| Broad beans; horse beans; dry | Pulses, Other | Legumes | 0.0 |
| Chick peas | Pulses, Other | Legumes | 0.0 |
| Cow peas; dry | Pulses, Other | Legumes | 0.0 |
| Pigeon peas | Pulses, Other | Legumes | 0.0 |
| Lentils | Pulses, Other | Legumes | 0.0 |
| Bambara beans | Pulses, Other | Legumes | 25.0 |
| Vetches | Pulses, Other | Legumes | 0.0 |
| Lupins | Pulses, Other | Legumes | 0.0 |
| Pulses; nes | Pulses, Other | Legumes | 0.0 |
| Flour of pulses | Pulses, Other | Legumes | 0.0 |
| Brazil nuts; with shell | Treenuts | Nuts & seeds | 49.0 |
| Cashew nuts; with shell | Treenuts | Nuts & seeds | 28.0 |
| Chestnuts | Treenuts | Nuts & seeds | 20.0 |
| Almonds; with shell | Treenuts | Nuts & seeds | 60.0 |
| Walnuts; with shell | Treenuts | Nuts & seeds | 65.5 |
| Pistachios | Treenuts | Nuts & seeds | 47.0 |
| Kolanuts | Treenuts | Nuts & seeds | 0.0 |
| Hazelnuts; with shell | Treenuts | Nuts & seeds | 59.0 |
| Areca nuts | Treenuts | Nuts & seeds | 0.0 |
| Nuts; nes | Treenuts | Nuts & seeds | 55.0 |
| Prepared nuts (exc. groundnuts) | Treenuts | Nuts & seeds | 4.7 |
| Soyabeans | Soyabeans | Legumes | 0.0 |
| Groundnuts (Shelled Eq) | Groundnuts (Shelled Eq) | Nuts & seeds | 0.0 |
| Sunflowerseed | Sunflowerseed | Nuts & seeds | 46.0 |
| Rape and Mustardseed | Rape and Mustardseed | Plant n-3 | 0.0 |
| Cottonseed | Cottonseed | PUFA | 0.0 |
| Coconuts - Incl Copra | Coconuts - Incl Copra | Fruits | 48.0 |
| Sesameseed | Sesameseed | Nuts & seeds | 0.0 |
| Palmkernels | Palmkernels | Saturated FA | 0.0 |
| Olives | Olives | PUFA | 0.0 |
| Oilcrops; Other | Oilcrops; Other | PUFA | 22.0 |
| Soyabean Oil | Soyabean Oil | PUFA | 0.0 |
| Groundnut Oil | Groundnut Oil | PUFA | 0.0 |
| Sunflowerseed Oil | Sunflowerseed Oil | PUFA | 0.0 |
| Rape and Mustard Oil | Rape and Mustard Oil | Plant n-3 | 0.0 |
| Cottonseed Oil | Cottonseed Oil | PUFA | 0.0 |
| Palmkernel Oil | Palmkernel Oil | Saturated FA | 0.0 |
| Palm Oil | Palm Oil | Saturated FA | 0.0 |
| Coconut Oil | Coconut Oil | Saturated FA | 0.0 |
| Sesameseed Oil | Sesameseed Oil | PUFA | 0.0 |
| Olive Oil | Olive Oil | PUFA | 0.0 |
| Ricebran Oil | Ricebran Oil | PUFA | 0.0 |
| Maize Germ Oil | Maize Germ Oil | PUFA | 0.0 |
| Oilcrops Oil; Other | Oilcrops Oil; Other | PUFA | 0.0 |
| Tomatoes | Tomatoes | Vegetables | 9.0 |
| Onions | Onions | Vegetables | 10.0 |
| Cabbages and other brassicas | Vegetables, Other | Vegetables | 25.9 |
| Artichokes | Vegetables, Other | Vegetables | 60.0 |
| Asparagus | Vegetables, Other | Vegetables | 47.0 |
| Lettuce and chicory | Vegetables, Other | Vegetables | 17.0 |
| Spinach | Vegetables, Other | Vegetables | 28.0 |
| Cassava leaves | Vegetables, Other | Vegetables | 0.0 |
| Cauliflowers and broccoli | Vegetables, Other | Vegetables | 50.0 |
| Pumpkins; squash; and gourds | Vegetables, Other | Vegetables | 21.3 |
| Cucumbers and gherkins | Vegetables, Other | Vegetables | 3.0 |
| Eggplants (aubergines) | Vegetables, Other | Vegetables | 19.0 |
| Chillies and peppers; green | Vegetables, Other | Vegetables | 16.6 |
| Onions (inc. shallots); green | Vegetables, Other | Vegetables | 17.0 |
| Garlic | Vegetables, Other | Vegetables | 13.0 |
| Leeks; other alliaceous veg. | Vegetables, Other | Vegetables | 28.0 |
| Beans; green | Vegetables, Other | Vegetables | 12.0 |
| Peas; green | Vegetables, Other | Vegetables | 34.0 |
| Leguminous vegetables; nes | Vegetables, Other | Vegetables | 3.0 |
| String beans | Vegetables, Other | Vegetables | 12.0 |
| Carrots and turnips | Vegetables, Other | Vegetables | 11.0 |
| Okra | Vegetables, Other | Vegetables | 14.0 |
| Maize; green | Vegetables, Other | Vegetables | 64.0 |
| Mushrooms and truffles | Vegetables, Other | Vegetables | 3.0 |
| Chicory roots | Vegetables, Other | Vegetables | 18.0 |
| Vegetables; fresh; nes | Vegetables, Other | Vegetables | 24.0 |
| Vegetables; dried; nes | Vegetables, Other | Vegetables | 2.1 |
| Vegetables; dehydrated | Vegetables, Other | Vegetables | 0.0 |
| Vegetables in vinegar | Vegetables, Other | Vegetables | 0.0 |
| Vegetables; preserved; nes | Vegetables, Other | Vegetables | 0.0 |
| Vegetables; frozen | Vegetables, Other | Vegetables | 3.1 |
| Vegetables in tem. preservatives | Vegetables, Other | Vegetables | 0.0 |
| Vegetables prepared or preserved; frozen | Vegetables, Other | Vegetables | 2.6 |
| Homogenous vegetables prepared | Vegetables, Other | Vegetables | 0.0 |
| Watermelons | Vegetables, Other | Vegetables | 48.0 |
| Other melons (inc. cantaloupes) | Vegetables, Other | Vegetables | 47.7 |
| Coffee substitutes; cont. coffee | Vegetables, Other | Vegetables | 0.0 |
| Oranges; Mandarines | Fruits, Other | Fruits | 25.3 |
| Lemons; Limes | Fruits, Other | Fruits | 31.5 |
| Grapefruit | Fruits, Other | Fruits | 47.0 |
| Citrus; Other | Fruits, Other | Fruits | 7.0 |
| Bananas | Fruits, Other | Fruits | 36.0 |
| Plantains | Fruits, Other | Fruits | 35.0 |
| Apples | Fruits, Other | Fruits | 10.0 |
| Pineapples | Fruits, Other | Fruits | 49.0 |
| Dates | Fruits, Other | Fruits | 9.0 |
| Grapes | Fruits, Other | Fruits | 16.7 |
| Pears | Fruits, Other | Fruits | 10.0 |
| Quinces | Fruits, Other | Fruits | 39.0 |
| Apricots | Fruits, Other | Fruits | 7.0 |
| Sour cherries | Fruits, Other | Fruits | 10.0 |
| Cherries | Fruits, Other | Fruits | 8.0 |
| Peaches and nectarines | Fruits, Other | Fruits | 6.5 |
| Plums and sloes | Fruits, Other | Fruits | 6.0 |
| Stone fruit; nes | Fruits, Other | Fruits | 7.3 |
| Pome fruit; nes | Fruits, Other | Fruits | 20.2 |
| Strawberries | Fruits, Other | Fruits | 6.0 |
| Raspberries | Fruits, Other | Fruits | 4.0 |
| Gooseberries | Fruits, Other | Fruits | 0.0 |
| Currants | Fruits, Other | Fruits | 2.0 |
| Blueberries | Fruits, Other | Fruits | 5.0 |
| Cranberries | Fruits, Other | Fruits | 2.0 |
| Berries; nes | Fruits, Other | Fruits | 0.8 |
| Figs | Fruits, Other | Fruits | 1.0 |
| Mangos; mangosteens; guavas | Fruits, Other | Fruits | 25.5 |
| Avocados | Fruits, Other | Fruits | 26.0 |
| Persimmons | Fruits, Other | Fruits | 17.0 |
| Cashewapple | Fruits, Other | Fruits | 0.0 |
| Kiwi fruit | Fruits, Other | Fruits | 25.0 |
| Papayas | Fruits, Other | Fruits | 38.0 |
| Fruit; tropical fresh; nes | Fruits, Other | Fruits | 30.5 |
| Fresh fruit; nes | Fruits, Other | Fruits | 25.0 |
| Fruit dried; nes | Fruits, Other | Fruits | 14.3 |
| Fruit juice; nes | Fruits, Other | Fruits | 0.0 |
| Fruit; prepared; nes | Fruits, Other | Fruits | 1.2 |
| Homogenized; cooked fruit prepared | Fruits, Other | Fruits | 0.0 |
| Coffee | Coffee | *none* | 0.0 |
| Cocoa Beans | Cocoa Beans | *none* | 0.0 |
| Tea | Tea | *none* | 0.0 |
| Pepper | Pepper | *none* | 0.0 |
| Pimento | Pimento | *none* | 0.0 |
| Cloves | Cloves | *none* | 0.0 |
| Vanilla | Spices, Other | *none* | 0.0 |
| Cinnamon (canella) | Spices, Other | *none* | 0.0 |
| Nutmeg; mace; and cardamoms | Spices, Other | *none* | 0.0 |
| Anise; badian; fennel; coriander | Spices, Other | *none* | 0.0 |
| Ginger | Spices, Other | *none* | 0.0 |
| Spices; nes | Spices, Other | *none* | 10.1 |
| Wine | Wine | *none* | 0.0 |
| Beer | Beer | *none* | 0.0 |
| Beverages; Fermented | Beverages; Fermented | *none* | 0.0 |
| Beverages; Alcoholic | Beverages; Alcoholic | *none* | 0.0 |
| Bovine Meat | Bovine Meat | Red meat | 19.0 |
| Mutton & Goat Meat | Mutton & Goat Meat | Red meat | 11.5 |
| Pigmeat | Pigmeat | Red meat | 18.0 |
| Poultry Meat | Poultry Meat | Red meat | 30.5 |
| Bird meat; nes | Meat, Other | Red meat | 9.4 |
| Horse meat | Meat, Other | Red meat | 0.0 |
| Meat of asses | Meat, Other | Red meat | 0.0 |
| Meat of mules | Meat, Other | Red meat | 0.0 |
| Camel meat | Meat, Other | Red meat | 0.0 |
| Rabbit meat | Meat, Other | Red meat | 0.0 |
| Meat of other rodents | Meat, Other | Red meat | 0.0 |
| Meat of other camelids | Meat, Other | Red meat | 0.0 |
| Game meat | Meat, Other | Red meat | 0.0 |
| Meat; dried; nes | Meat, Other | Red meat | 0.0 |
| Meat; nes | Meat, Other | Red meat | 6.7 |
| Snails; not sea | Meat, Other | Red meat | 0.0 |
| Offals of cattle; edible | Offals, Edible | Red meat | 10.7 |
| Offals of sheep; edible | Offals, Edible | Red meat | 8.4 |
| Offals of goats; edible | Offals, Edible | Red meat | 0.0 |
| Offals of pigs; edible | Offals, Edible | Red meat | 6.4 |
| Offals; liver; chicken | Offals, Edible | Red meat | 0.0 |
| Offals; liver; geese | Offals, Edible | Red meat | 0.0 |
| Offals; liver; duck | Offals, Edible | Red meat | 0.0 |
| Offals; nes | Offals, Edible | Red meat | 7.8 |
| Butter; cow milk | Butter, Ghee | Milk | 0.0 |
| Ghee; butteroil of cow milk | Butter, Ghee | Milk | 0.0 |
| Butter of buffalo milk | Butter, Ghee | Milk | 0.0 |
| Ghee oil of buffalo milk | Butter, Ghee | Milk | 0.0 |
| Butter; ghee of sheep milk | Butter, Ghee | Milk | 0.0 |
| Cream | Butter, Ghee | Milk | 0.0 |
| Fats; Animals; Raw | Fats; Animals; Raw | Saturated FA | 0.0 |
| Fish; Body Oil | Fish; Body Oil | Seafood n-3 | 0.0 |
| Fish; Liver Oil | Fish; Liver Oil | Seafood n-3 | 0.0 |
| Hen eggs; in shell | Eggs | *none* | 12.0 |
| Eggs; liquid | Eggs | *none* | 12.0 |
| Eggs; dried | Eggs | *none* | 0.0 |
| Other bird eggs; in shell | Eggs | *none* | 11.3 |
| Cow milk; whole; fresh | Milk – Excluding Butter | Milk | 0.0 |
| Buffalo milk; whole; fresh | Milk – Excluding Butter | Milk | 0.0 |
| Sheep milk; whole; fresh | Milk – Excluding Butter | Milk | 0.0 |
| Goat milk; whole; fresh | Milk – Excluding Butter | Milk | 0.0 |
| Camel milk; whole; fresh | Milk – Excluding Butter | Milk | 0.0 |
| Product of natural milk constit. | Milk – Excluding Butter | Milk | 0.0 |
| Ice cream and edible ice | Milk – Excluding Butter | Milk | 0.0 |
| Freshwater Fish | Freshwater Fish | Seafood | 0.0 |
| Demersal Fish | Demersal Fish | Seafood | 0.0 |
| Pelagic Fish | Pelagic Fish | Seafood | 0.0 |
| Marine Fish; Other | Marine Fish; Other | Seafood | 0.0 |
| Crustaceans | Crustaceans | Seafood | 0.0 |
| Cephalopods | Cephalopods | Seafood | 0.0 |
| Molluscs; Other | Molluscs; Other | Seafood | 0.0 |
| Aquatic Animals; Others | Aquatic Animals; Others | Seafood | 0.0 |
| Aquatic Plants | Aquatic Plants | Vegetables | 0.0 |
| Miscellaneous + (Total) | Miscellaneous + (Total) | *none* | 0.0 |
| Wheat Flour | Wheat | Whole grains | 0.0 |
| Corn Flour | Maize | Whole grains | 0.0 |
| Millet Flour | Millet | Whole grains | 0.0 |
| Sorghum Flour | Sorghum | Whole grains | 0.0 |

**S1 Table. Associated GDD food groups and non-edible (discarded) percentage for each food studied**
