# Supplementary material for: Global Expanded Nutrient Supply (GENuS) Model: A New Method for Estimating the Global Dietary Supply of Nutrients
Source: PLoS One. 2016 Jan 25;11(1):e0146976. doi: 10.1371/journal.pone.0146976 (PMC4726504; doi:10.1371/journal.pone.0146976)
Supplement: S2 Table — (DOCX) [file pone.0146976.s004.docx]

| Nutrient(s) | Food Vehicles |
| --- | --- |
| Iron | Wheat flour, corn flour/meal, rice |
| Zinc | Wheat flour, corn flour/meal, rice |
| Vitamin A | Wheat flour, corn flour/meal, rice, milk, sugar, cooking oil, margarine |
| Thiamin (B1) | Wheat flour, corn flour/meal, rice |
| Riboflavin (B2) | Wheat flour, corn flour/meal, rice |
| Niacin (B3) | Wheat flour, corn flour/meal, rice |
| Folic acid | Wheat flour, corn flour/meal, rice |

**S2 Table. Fortified nutrients studied and their associated food vehicles**
